# Lecanemab use in Chinese patients with Alzheimer's disease: a 12‐month multicenter real‐world study

**DOI:** 10.1002/alz.71702

**Published:** 2026-08-06

**Authors:** Hao Wu, Qin Chen, Min Fang, Huidong Tang, Huayan Liu, Jun Liu, Jiewen Zhang, Lijun Chi, Shuai Liu, Jiawei Xin, Lumin Leng, Pan Wang, Song Chi, Yi Li, Jianwen Chen, Ligong Zhang, Jinhong Zhang, Qiang Ma, Xuezhen Wang, Xinling Meng, Jianfei Nao, Xiangqing Li, Yan Lv, Yanhong Jia, Qing Zhao, Chunyan Liu, Jinghuan Gan, Jing Zhu, Yehua Song, Huaian Li, Min Fei, Xia Guo, Jingwen Liu, Guoping Peng, Xiaochun Chen, Yong Ji

**Affiliations:** ^1^ Department of Neurology, Tianjin Key Laboratory of Cerebral Vascular and Neurodegenerative Diseases Huanhu Hospital Affiliated to Tianjin Medical University Tianjin China; ^2^ Department of Neurology West China Hospital of Sichuan University Chengdu Sichuan China; ^3^ Department of Neurology Shanghai Tongren Hospital, Shanghai Jiao Tong University School of Medicine Shanghai China; ^4^ Department of Geriatrics, Medical Center on Aging Ruijin Hospital, Shanghai Jiao Tong University School of Medicine Shanghai China; ^5^ Department of Neurology First Hospital of China Medical University Shenyang Liaoning China; ^6^ Department of Neurology, Institute of Neuroscience Key Laboratory of Neurogenetics and Channelopathies of Guangdong Province and the Ministry of Education of China The Second Affiliated Hospital, Guangzhou Medical University Guangzhou Guangdong China; ^7^ Department of Neurology Zhengzhou University People's Hospital Henan University People's Hospital Henan Provincial People's Hospital Zhengzhou Henan China; ^8^ Department of Neurology The First Affiliated Hospital of Harbin Medical University Harbin Heilongjiang China; ^9^ Department of Neurology Fujian Medical University Union Hospital Fujian Key Laboratory of Molecular Neurology and Institute of Neuroscience Fujian Medical University Fuzhou Fujian China; ^10^ Department of Neurology The First Affiliated Hospital, Zhejiang University School of Medicine Hangzhou Zhejiang China; ^11^ Department of Neurology The Affiliated Hospital of Qingdao University Qingdao Shandong China; ^12^ Department of Neurology Qilu Hospital, Shandong University Jinan Shandong China; ^13^ Department of Neurology The First Affiliated Hospital of Dalian Medical University Dalian Liaoning China; ^14^ Department of Neurology Shengli Oilfield Central Hospital Dongying Shandong China; ^15^ Department of Neurology Cangzhou People's Hospital Cangzhou Hebei China; ^16^ Department of Neurology Affiliated Zhongshan Hospital of Dalian University Dalian Liaoning China; ^17^ Department of Neurology Affiliated Hospital of Binzhou Medical University Binzhou Shandong China; ^18^ Department of Brain Disease Affiliated Traditional Chinese Medicine Hospital of Xinjiang Medical University Urumqi Xinjiang China; ^19^ Department of Neurology Shengjing Hospital of China Medical University Shenyang Liaoning China; ^20^ Department of Neurology Zibo Central Hospital Zibo Shandong China; ^21^ Department of Neurology Hainan General Hospital Haikou Hainan China; ^22^ Department of Neurology Baotou Central Hospital Baotou Inner Mongolia China; ^23^ Department of Neurology China‐Japan Union Hospital of Jilin University Changchun Jilin China; ^24^ Department of Neurology Aviation General Hospital Beijing China; ^25^ Department of Neurology Beijing Friendship Hospital, Capital Medical University Beijing China; ^26^ Department of Neurology Linfen Central Hospital Linfen Shanxi China; ^27^ Department of Neurology The Second Affiliated Hospital of Xiamen Medical College Xiamen Fujian China; ^28^ Department of Geriatrics Qinhuangdao Hospital of Dongfang Hospital, Beijing University of Chinese Medicine, Qinhuangdao Hospital of Traditional Chinese Medicine Qinhuangdao Hebei China; ^29^ Department of Neurology Yuncheng Central Hospital Yuncheng Shanxi China; ^30^ Department of Neurology The First Affiliated Hospital of Baotou Medical College Baotou Inner Mongolia China; ^31^ Department of Neurology Shanghai Tenth People's Hospital, Tongji University School of Medicine Shanghai China

**Keywords:** Alzheimer's disease, amyloid‐related imaging abnormalities, blood‐based biomarkers, efficacy, lecanemab, real‐world study

## Abstract

**INTRODUCTION:**

We evaluated lecanemab's safety and cognitive outcomes in Chinese patients with Alzheimer's disease (AD) and the utility of blood‐based biomarkers (BBMs) for treatment guidance.

**METHODS:**

A multicenter, real‐world cohort enrolled 1042 patients receiving lecanemab, with 453, 359, 97 patients followed up at 3, 6, 12 months, respectively. Safety outcomes included amyloid‐related imaging abnormalities (ARIA) and infusion‐related reactions (IRRs), and the main *clinical* outcome was Clinical Dementia Rating Scale–Sum of Boxes (CDR‐SB) score change.

**RESULTS:**

Of 1,042 patients, ARIA occurred in 7.87%, and IRRs in 16.12%. Discontinuation was 16.51%, mainly due to financial burden. CDR‐SB did not change at 12 months overall or in any subgroup. High‐accuracy BBMs were the sole AD biomarker assay in 5.28%, with comparable outcomes.

**DISCUSSION:**

Lecanemab exhibited *favorable* 12‐month safety, with no clear evidence of cognitive decline, and BBMs hold potential for treatment guidance. Further studies using a control group are now warranted.

## Highlights

- This study provides real‐world evidence on the 12‐month safety and cognitive outcomes of lecanemab in a large Chinese cohort.
- It offers an initial real‐world assessment of high‐accuracy blood‐based biomarkers for guiding lecanemab treatment.
- The findings suggest a potential biomarker‐informed pathway for disease‐modifying therapy, which warrants further validation.

## 1 BACKGROUND

Alzheimer's disease (AD) is the leading cause of dementia worldwide.^1^, ^2^, ^3^ China bears a disproportionate share of this burden with an estimated 16.99 million people currently living with AD and related dementias, contributing nearly 30% of global cases. This represents a substantial public health burden with profound socioeconomic implications.

For decades, available treatments for AD were limited to symptomatic management. Guided by the amyloid‐beta (Aβ) hypothesis, drug development has led to a novel class of disease‐modifying therapies, including anti‐Aβ monoclonal antibodies.^4^, ^5^, ^6^, ^7^ The phase III Clarity AD trial of lecanemab, the first anti‐Aβ monoclonal antibody approved for use in China, demonstrated a 27% reduction in clinical decline on the Clinical Dementia Rating–Sum of Boxes (CDR‐SB) in patients with early AD when compared with placebo.^8^ Post‐marketing experience, however, has highlighted safety concerns, particularly infusion‐related reactions (IRRs) and amyloid‐related imaging abnormalities (ARIA).^9^, ^10^, ^11^

Translating lecanemab from rigorously controlled trials to routine clinical practice introduces uncertainties, and its clinical performance in real‐world settings remains insufficiently characterized. Patients encountered in routine clinical practice often present with a broader age range, more comorbidities, and less frequent radiologic monitoring than those enrolled in trials. Moreover, variations in clinicians’ experience with anti‐Aβ therapies may influence ARIA detection, the timing of interventions, and ultimately patient outcomes. These population‐specific factors underscore the need to clarify lecanemab's efficacy and safety profile in Chinese patients to guide risk–benefit considerations and support more tailored clinical recommendations.

Blood‐based biomarkers (BBMs) have recently emerged as promising alternatives for detecting AD pathology.^12^, ^13^, ^14^ Several BBMs, including Aβ_42_/Aβ_40_ and tau phosphorylated at different sites (such as p‐tau181, p‐tau217, p‐tau205, and p‐tau231), show strong correlations with underlying AD pathology.^15^, ^16^, ^17^ Compared with amyloid positron emission tomography (PET) and cerebrospinal fluid (CSF) testing, BBMs are less costly, more accessible, and often more acceptable to patients. Their practicality is particularly relevant given the increasing diagnostic demands associated with Aβ‐targeting therapies such as lecanemab.^18^ The use of BBMs to diagnose AD pathology and guide lecanemab treatment, however, remains insufficiently validated in real‐world settings, and the generalizability of BBM‐based diagnostics and their correlation with long‐term treatment outcomes are unclear.

We conducted this multicenter real‐world study to address these evidence gaps by evaluating the 12‐month safety and clinical outcomes of lecanemab in Chinese patients with AD and examining the utility of BBMs for diagnosing AD pathology and guiding treatment initiation. We sought to provide evidence‐based support for clinical decision‐making, risk mitigation strategies, and the integration of non‐invasive BBMs into lecanemab treatment workflows in Chinese clinical practice.

## 2 METHODS

### 2.1 Study design and data source

This multicenter, observational cohort study was conducted across 37 tertiary academic medical centers in China designated as national clinical research centers for neurological diseases, Level 3 Grade A teaching hospitals, or regional cognitive disorder centers. These sites span seven geographic regions (North, Northeast, East, South, Central, Southwest, and Northwest China) to ensure national representativeness. Data were extracted from electronic medical records using a standardized, piloted case report form. Trained neurology residents or research coordinators at each site collected data under unified protocols. Tianjin Huanhu Hospital served as the coordinating center and convened regular meetings to ensure data consistency and quality control. The study flowchart is shown in **eFigure** **1**.

The study protocol was approved by the institutional review board of Tianjin Huanhu Hospital, which acted as the central ethics committee (ID: 2025‐305). Informed consent was obtained from all participants or their legal representatives. All participating sites provided institutional endorsement. The study adhered to the STROBE reporting guidelines for observational studies.

**RESEARCH IN CONTEXT**

1. Systematic review: The authors reviewed the literature using PubMed. While lecanemab has demonstrated efficacy in early Alzheimer's disease (AD) in clinical trials, its real‐world safety and 12‐month clinical outcomes, particularly in Chinese patient populations, remain incompletely characterized. The recent integration of high‐accuracy (≥90% sensitivity and specificity) blood‐based biomarkers (BBMs) into diagnostic guidelines highlights the need to evaluate their clinical utility in guiding lecanemab treatment in routine practice.
2. **Interpretation**: Our findings provide preliminary observational evidence indicating a manageable safety profile and no statistically significant cognitive decline over 12 months of lecanemab treatment in routine clinical practice in China. These data also offer early real‐world support for the use of high‐accuracy BBMs to identify amyloid pathology and inform treatment decisions. The relatively high rate of treatment discontinuation, largely driven by financial constraints, highlights an important barrier to access that warrants consideration. Overall, these findings contribute real‐world evidence to the evolving understanding of disease‐modifying therapies for AD.
3. **Future directions**: Future research should prioritize prospective, longitudinal studies in larger Chinese cohorts to better characterize long‐term clinical outcomes and safety. Further validation of BBMs across screening, diagnosis, monitoring, and outcome prediction is needed. In parallel, health policy and care pathway development will be important to address treatment affordability and support equitable access to lecanemab for patients with AD.

### 2.2 Patient population

This study included patients with AD who initiated at least one infusion of lecanemab between June 26, 2024, and November 1, 2025, at any participating center. Inclusion criteria were: age ≥ 40 years; both sexes; AD dementia meeting the 2024 Alzheimer's Association diagnostic criteria^19^; documented Aβ‐positivity confirmed by Aβ‐PET, CSF, or BBMs with sensitivity and specificity ≥ 90%; and a clinical decision to initiate lecanemab treatment. Exclusion criteria were: dementia of non‐AD etiologies; severe comorbidities; history of substance abuse or mental disorders; and inability to complete cognitive assessments. For patients with conditions for which lecanemab is generally not recommended according to the Clarity AD trial,^8^ such as moderate dementia, clinicians informed patients of the reasons why lecanemab may be unsuitable and the associated risks. For those who insisted on lecanemab treatment, two neurologists conducted a more comprehensive evaluation and engaged in detailed discussions with the patients and their caregivers.

### 2.3 Biomarker assessments and apolipoprotein E genotyping

Because of the real‐world, multicenter design, amyloid pathology status was confirmed at each participating tertiary academic medical center using standardized clinical protocols rather than a central laboratory. Aβ‐PET imaging was performed using either 11C‐labeled Pittsburgh Compound B or 18F‐florbetapi radiotracers. CSF Aβ_42_/Aβ_40_ ratios and p‐tau181 were quantified using validated automated immunoassays in hospital‐certified laboratories. Plasma p‐tau217 and Aβ_42_/Aβ_40_ were measured using high‐sensitivity single‐molecule immunoassays, with sensitivity and specificity ≥ 90%. Apolipoprotein E (ApoE) genotype was determined using validated polymerase chain reaction‐based methods.

### 2.4 Treatment and clinical monitoring

All patients received standard intravenous infusions of lecanemab (10 mg/kg) every 2 weeks in accordance with the approved prescribing information. Treating physicians could interrupt or modify dosing based on individual clinical circumstances.

Cognitive and functional assessments, including the CDR‐SB,^20^ Mini‐Mental State Examination (MMSE)^21^, Clinical Dementia Rating‐Global Score (CDR‐GS),^22^ and Activities of Daily Living (ADL) scale,^23^, ^24^ and other assessments were performed at baseline and during follow‐up according to routine practice at each center, typically at 3, 6, and 12 months.

Magnetic resonance imaging (MRI) monitoring was performed using 3.0 T or higher‐field scanners. To promote harmonization of image acquisition and interpretation across sites, all participating radiologists received standardized training. Baseline MRI results were available for all patients, and baseline microhemorrhages, superficial siderosis, and Fazekas scores were determined from these scans. Follow‐up MRI was usually conducted at the 5th to 7th infusion (approximately 3 to 4 months) and at the 14th infusion (approximately 7 months). Additional scans were obtained if clinical symptoms emerged. All MRI examinations included fluid‐attenuated inversion recovery sequences to evaluate ARIA–edema/effusion (ARIA‐E) and T2* gradient‐recalled echo or susceptibility‐weighted imaging sequences to evaluate ARIA–hemorrhage/hemosiderin deposition (ARIA‐H). All imaging findings suggestive of ARIA were reviewed and confirmed by a dedicated panel of radiologists and clinical experts.

### 2.5 Adverse event identification and definition

Adverse events, including ARIA and IRRs, were identified through review of physician and nursing documentation in inpatient and outpatient electronic health records. The timing, severity, and clinical outcomes of all events were extracted from the medical records. When documentation was incomplete or unclear, information was verified with the patient or caregiver. All suspected ARIA events were confirmed by neuroradiologic review.

### 2.6 Statistical analyses and data visualization

All statistical analyses were performed using SAS 9.4. Graphs were generated with Microsoft Excel and R 4.3.1. A two‐sided P < 0.05 was considered statistically significant. No imputation was performed for any missing data in this study. Complete case analysis was used for all longitudinal cognitive assessments. Differences between groups were assessed using the t‐test for normally distributed continuous variables and the Wilcoxon rank‐sum test for non‐normally distributed continuous variables. For categorical variables, the chi‐squared test or Fisher's exact test was applied. The relationship between the occurrence of ARIA and various risk factors was analyzed using a logistic regression model, which was adjusted for age, sex, weight, CDR‐SB, ApoE ε4 genotype, baseline microhemorrhage count, and baseline superficial siderosis. For longitudinal changes in cognitive scale scores across baseline, 3, 6, and 12 months, an analysis of covariance (ANCOVA) model was applied. The model included respective baseline scores, age, and years of education as adjustment covariates to estimate adjusted least squares means.

## 3 RESULTS

### 3.1 Characteristics of patients receiving lecanemab

Between June 26, 2024, and November 1, 2025, a total of 1,042 patients (mean [SD] age, 68.93 [8.60] years; 661 [63.44%] female) who initiated lecanemab treatment were included in the final analysis. ApoE genotype status was available for most participants; ApoE ε4 carriers (ε2/ε4, ε3/ε4, and ε4/ε4 genotypes) accounted for 516 patients (49.52%). At baseline, neuroimaging markers showed that 173 participants (16.60%) had 1‐4 cerebral microbleeds and 13 (1.25%) had ≥5 microbleeds. Superficial siderosis was present in 18 participants (1.73%). Vascular risk factors were common; 83 (7.97%) were taking antiplatelet agents, and 8 (0.77%) were receiving anticoagulants. Most patients underwent PET alone (628 [60.27%]) to confirm Aβ positivity. In 55 patients (5.28%), a high‐accuracy blood‐based biomarker test was the sole assay performed. Isolated CSF testing was uncommon (27 [2.59%]) (**Table** **1**). Demographic characteristics (age, sex, weight, ApoE status) and daily function scores (CDR‐SB, ADL) were generally comparable between the BBM‐only and non‐BBM diagnostic groups (eTable 1 in Supplement 1). A total of 453, 359, and 97 patients completed the 3‐, 6‐, and 12‐month follow‐up visits, respectively (eFigure 1 in Supplement 1).

**TABLE 1:** Baseline characteristics, comorbidities, medication use, and biomarkers of study participants.

Characteristic	Total
Participants, No.	1042
Age, mean (SD), y	68.93 (8.60)
Sex, no. (%)	
Male	381 (36.56)
Female	661 (63.44)
Education, mean (SD), y	11.35 (4.02)
Height, mean (SD), cm	163.60 (8.38)
Weight, mean (SD), kg	59.95 (9.95)
MMSE score, mean (SD)	19.94 (4.97)
MoCA score, mean (SD)	15.51 (5.26)
CDR‐GS, No. (%)	
0.5	609 (58.45)
1	262 (25.14)
2	55 (5.28)
3	9 (0.86)
Unknown	107 (10.27)
CDR‐SB score, mean (SD)	3.82 (2.81)
ADL score, mean (SD)	25.32 (6.73)
Total NPI score, median (Q1, Q3)	2 (0,8)
NPI caregiver score, median (Q1, Q3)	3 (0,6)
HAMD score, median (Q1, Q3)	3 (1,7)
HAMA score, median (Q1, Q3)	4 (1,7)
ApoE status, No. (%)	
ε2/ε3	73 (7.01)
ε3/ε3	394 (37.81)
ε2/ε4	45 (4.32)
ε3/ε4	378 (36.28)
ε4/ε4	93 (8.93)
Unknown	59 (5.66)
No. of superficial siderosis, No. (%)	
0	1024 (98.27)
1	18 (1.73)
No. of microhemorrhages, No. (%)	
0	856 (82.15)
1‐4	173 (16.60)
≥5	13 (1.25)
Fazekas score, No. (%)	
0	88 (8.45)
1	447 (42.90)
2	156 (14.97)
3	28 (2.69)
Unknown	323 (31.00)
Comorbidities, No. (%)	
Hypertension	291 (27.93)
Diabetes	170 (16.31)
Hyperlipidemia	172 (16.51)
Coronary heart disease	83 (7.97)
Atrial fibrillation	22 (2.11)
Cerebral infarction	48 (4.61)
Medication use, No. (%)	
Cholinesterase inhibitor	618 (59.31)
Memantine	263 (25.24)
Anticoagulant	8 (0.77)
Antiplatelet	83 (7.97)
Antihypertensive	243 (23.32)
Antidiabetic agents	148 (14.20)
Diagnostic biomarkers, No. (%)	
PET‐only	628 (60.27)
BBMs‐only	55 (5.28)
CSF‐only	27 (2.59)
BBMs and PET	263 (25.24)
PET and CSF	37 (3.55)
BBMs and CSF	7 (0.67)
BBMs, PET, and CSF	25 (2.40)

Abbreviations: ADL, activities of daily living; ApoE, apolipoprotein E; BBMs, blood‐based biomarkers; CDR‐GS, Clinical Dementia Rating‐Global Score; CDR‐SB, Clinical Dementia Rating‐Sum of Boxes; CSF, cerebrospinal fluid; HAMA, Hamilton Anxiety Rating Scale; HAMD, Hamilton Depression Rating Scale; MMSE, Mini‐Mental State Examination; MoCA, Montreal Cognitive Assessment; NPI, Neuropsychiatric Inventory; PET, positron emission tomography.

### 3.2 Occurrence, related factors, and prognosis of ARIA

A total of 82 participants (7.87%) developed ARIA during follow‐up, including 30 (2.88%) with ARIA‐E and 65 (6.24%) with ARIA‐H; 11 patients (1.06%) had symptomatic ARIA. Any ARIA occurrence was significantly associated with ApoE ε4 carriage, cerebral microhemorrhages at baseline, and superficial siderosis at baseline (all *P* < 0.001). By contrast, we did not observe significant associations with age, sex, educational level, vascular comorbidities, use of anticoagulants or antiplatelet agents, or Fazekas score (eTable 2 in Supplement 1). Additional details regarding ARIA are available in eTable 3 and eTable 4 in Supplement 1. Among the 30 participants with ARIA‐E, 26 (2.50% of the full cohort) were asymptomatic, and 4 (0.38%) experienced transient headache, dizziness, slowed responses, or mild visual disturbance. Among those with ARIA‐H, 58 (5.57%) were asymptomatic and 7 (0.67%) reported similar mild symptoms. We did not identify significant differences in baseline characteristics, including age, sex, disease severity, body weight, or ApoE genotype in symptomatic and asymptomatic patients with ARIA‐E (*P* > 0.05), and findings were similar for those with ARIA‐H.

Logistic regression models for any ARIA, ARIA‐E, and ARIA‐H, adjusted for age, sex, weight, CDR‐SB, ApoE ε4 genotype, baseline microhemorrhage count, and superficial siderosis showed that baseline presence of superficial siderosis (adjusted odds ratio [OR], 8.52; 95% confidence interval [CI], 2.39–30.35; *P* < 0.001), five or more microhemorrhages (adjusted OR, 8.27; 95% CI, 2.06–33.22; *P *= 0.003), one to four microhemorrhages (adjusted OR, 2.38; 95% CI, 1.33–4.27; *P* = 0.004), and homozygous ApoE ε4 genotype (adjusted OR, 3.95; 95% CI, 1.86–8.41; *P* < 0.001) were independently associated with any ARIA. Age, sex, weight, and CDR‐SB were not significantly associated (**Table** **2**).

**TABLE 2:** Multivariable logistic regression predictors of amyloid‐related imaging abnormalities.

	Any ARIA		ARIA‐E		ARIA‐H	
Variable	Odds ratio (95% CI)	*p‐*value	Odds ratio (95% CI)	*p‐*value	Odds ratio (95%CI)	*p‐*value
Age	1.01 (0.98–1.04)	0.489	0.95 (0.91–1.00)	0.042	1.03 (1.00–1.07)	0.081
Sex						
Male	Reference		Reference		Reference	
Female	0.89 (0.48–1.67)	0.726	0.94 (0.33–2.72)	0.913	0.95 (0.47–1.93)	0.891
Weight	0.98 (0.95–1.01)	0.141	1.00 (0.95–1.06)	0.913	0.96 (0.93–1.00)	0.029
CDR‐SB	1.01 (0.92–1.11)	0.78	0.91 (0.75–1.09)	0.290	1.03 (0.93–1.15)	0.522
ApoE ε4 status						
ε4 noncarrier	Reference		Reference		Reference	
ε4 heterozygous	1.45 (0.81–2.60)	0.210	1.03 (0.39–2.77)	0.948	1.85 (0.95–3.60)	0.072
ε4 homozygous	3.95 (1.86–8.41)	<0.001	2.51 (0.72–8.74)	0.149	5.03 (2.17–11.69)	<0.001
Microhemorrhages						
0	Reference		Reference		Reference	
1–4	2.38 (1.33–4.27)	0.004	0.91 (0.26–3.23)	0.883	2.82 (1.50–5.29)	0.001
≥5	8.27 (2.06–33.22)	0.003	9.78 (1.79–53.34)	0.008	13.95 (3.44–56.64)	<0.001
Superficial siderosis						
0	Reference		Reference		Reference	
1	8.52 (2.39‐30.35)	<0.001	19.34 (4.32–86.61)	<0.001	2.19 (0.40–11.91)	0.364

*Note*: The logistic regression model was adjusted for age, sex, weight, CDR‐SB, ApoE genotype, baseline microhemorrhage count, and baseline superficial siderosis.

Abbreviations: ApoE, apolipoprotein E; ARIA, amyloid‐related imaging abnormality; ARIA‐E, amyloid‐related imaging abnormality–edema/effusion; ARIA‐H, amyloid‐related imaging abnormality–hemorrhage/hemosiderin deposition; CDR‐SB, Clinical Dementia Rating–Sum of Boxes; CI, confidence interval.

**FIGURE 1:**
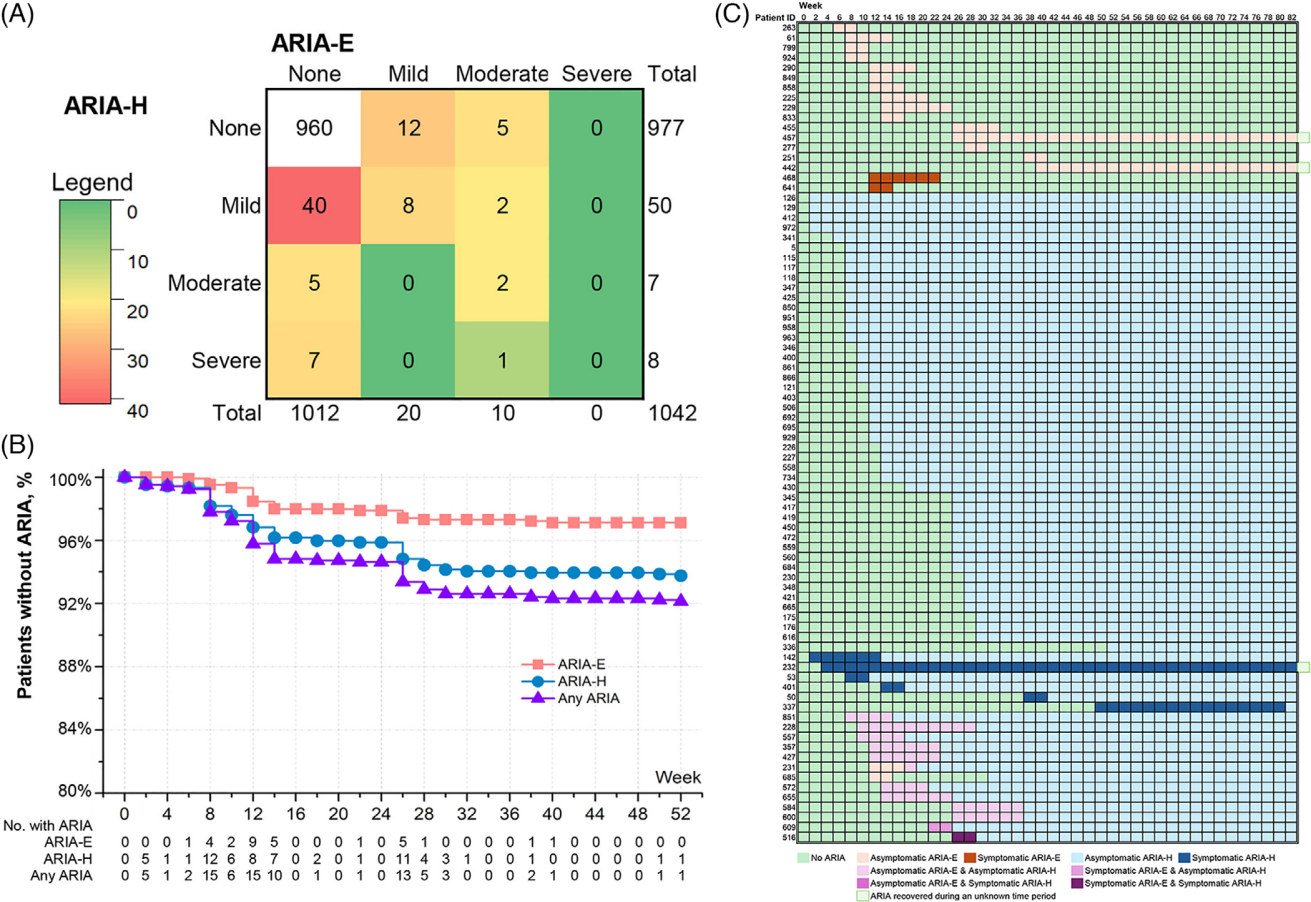
Distribution, temporal trajectory, and longitudinal clinical course of ARIA. (A) Distribution of patients across four severity categories (none, mild, moderate, severe) for ARIA‐H and ARIA‐E. Color gradient represents the relative proportion of patients in each category, with warmer colors indicating higher proportions and cooler colors indicating lower proportions. (B) Temporal pattern of ARIA onset over the follow‐up period. Proportion of patients is plotted at 2‐week intervals for ARIA‐E, ARIA‐H, and any ARIA. (C) Longitudinal ARIA profile for each participant. Weekly ARIA status is displayed using color‐coded cells representing the presence and type of ARIA. ARIA, amyloid‐related imaging abnormality; ARIA‐E, amyloid‐related imaging abnormality–edema/effusion; ARIA‐H, amyloid‐related imaging abnormality–hemorrhage/hemosiderin deposition

Of the 82 ARIA cases, 60 (73.17%) were mild (Figure 1A, eTable 4 in Supplement 1). Isolated ARIA‐H occurred in 52 participants (4.99%), including 40 (3.84%) with mild severity, 5 (0.48%) with moderate severity, and 7 (0.67%) with severe severity. No participants experienced severe ARIA‐E. ARIA‐H emerged as early as week 2 and ARIA‐E by week 6, with overall peak incidence between weeks 8 and 14 (Figure 1B). After week 28, new cases were infrequent. Among the 30 patients with ARIA‐E, asymptomatic ARIA‐E were rare occurrences and typically resolved spontaneously within 4 to 12 weeks. Symptomatic ARIA‐H generally resolved by week 24. Of note, we did not observe progression from asymptomatic to symptomatic ARIA after this point (Figure 1C).

### 3.3 Occurrence and prognosis of IRRs

**FIGURE 2:**
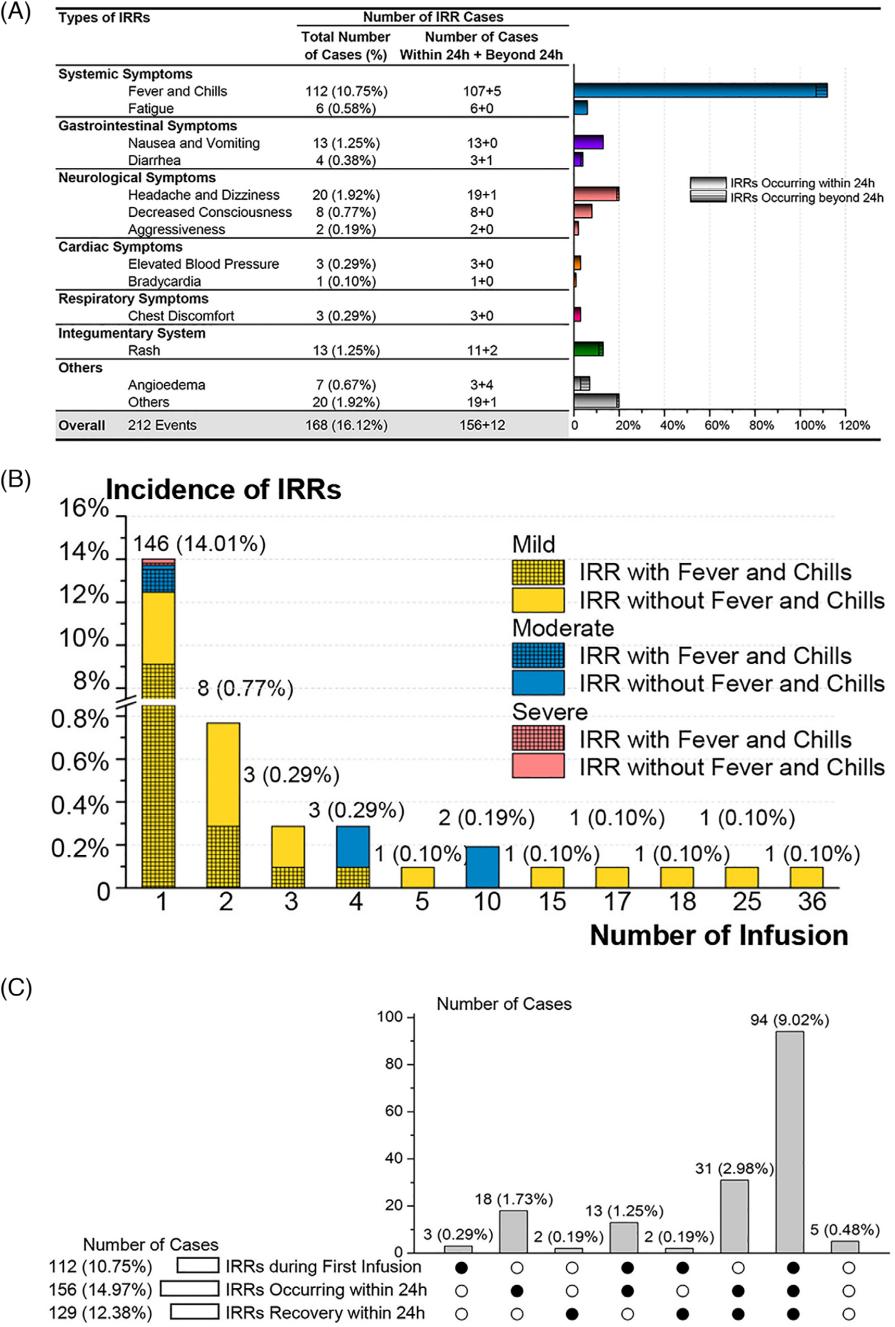
Clinical profile of IRRs: manifestation, incidence by infusion cycle, and onset/recovery timing. (A) Clinical manifestations of IRRs and proportion of cases occurring within and beyond 24 hours after infusion. (B) Incidence of IRRs by infusion number, showing the proportion of patients with IRRs at each infusion and stratified by severity (mild, moderate, severe). (C) Onset and recovery timing of IRRs; solid circles indicate event presence, empty circles indicate absence, vertical bars represent case counts, and horizontal bars represent total cases per category. IRRs, infusion‐related reactions

Among the 1,042 participants who received lecanemab, 168 (16.12%) experienced at least one IRR (**Figure** **2**). Most events (92.86%) occurred within 24 hours of infusion. Fever or chills were the most common IRR (112 cases, 10.75%), with 107 of these occurring within 24 hours. Notably, no IRRs required hospitalization. Delayed events with onset > 24 hours after infusion were rare, but included isolated cases of fever or chills, diarrhea, rash, facial edema, and headache (Figure 2A). The highest incidence of IRRs occurred during the first infusion, affecting 146 of 1,042 patients (14.01%), and most were mild (138 of 146, 94.52%). The incidence declined with subsequent infusions: 0.77% (8 of 1,042) during the second infusion and 0.29% (3 of 1,042) from the third infusion onwards (Figure 2B). Overall, 129 of 168 IRRs (76.79%) resolved spontaneously within 24 hours (Figure 2C). The incidence and severity of IRRs by infusion number are summarized in eTable 5 in Supplement 1.

### 3.4 Discontinuation of lecanemab

A total of 172 participants discontinued treatment during the study period (eTable 6, eTable 7, eFigure 2 in Supplement 1 **)**. The most frequent reason was financial burden (40 participants, 23.26%), followed by other reasons (37 participants, 21.51%) and ARIA (27 participants, 15.70%). Adverse event‐related discontinuation, which included asymptomatic ARIA, symptomatic ARIA, and infusion‐related reactions, accounted for 55 of 172 cases (31.98%), with asymptomatic ARIA representing the most prevalent adverse event‐related cause. Most discontinuations occurred during the early infusion phase.

### 3.5 Cognitive monitoring

Data in eTable 8 in Supplement 1 indicate that the sample size decreased progressively during follow‐up, with 453 patients assessed at 3 months, 359 at 6 months, and 97 at 12 months. Age, sex, and educational level at follow‐up were comparable to baseline, confirming that participant characteristics remained relatively stable.

**FIGURE 3:**
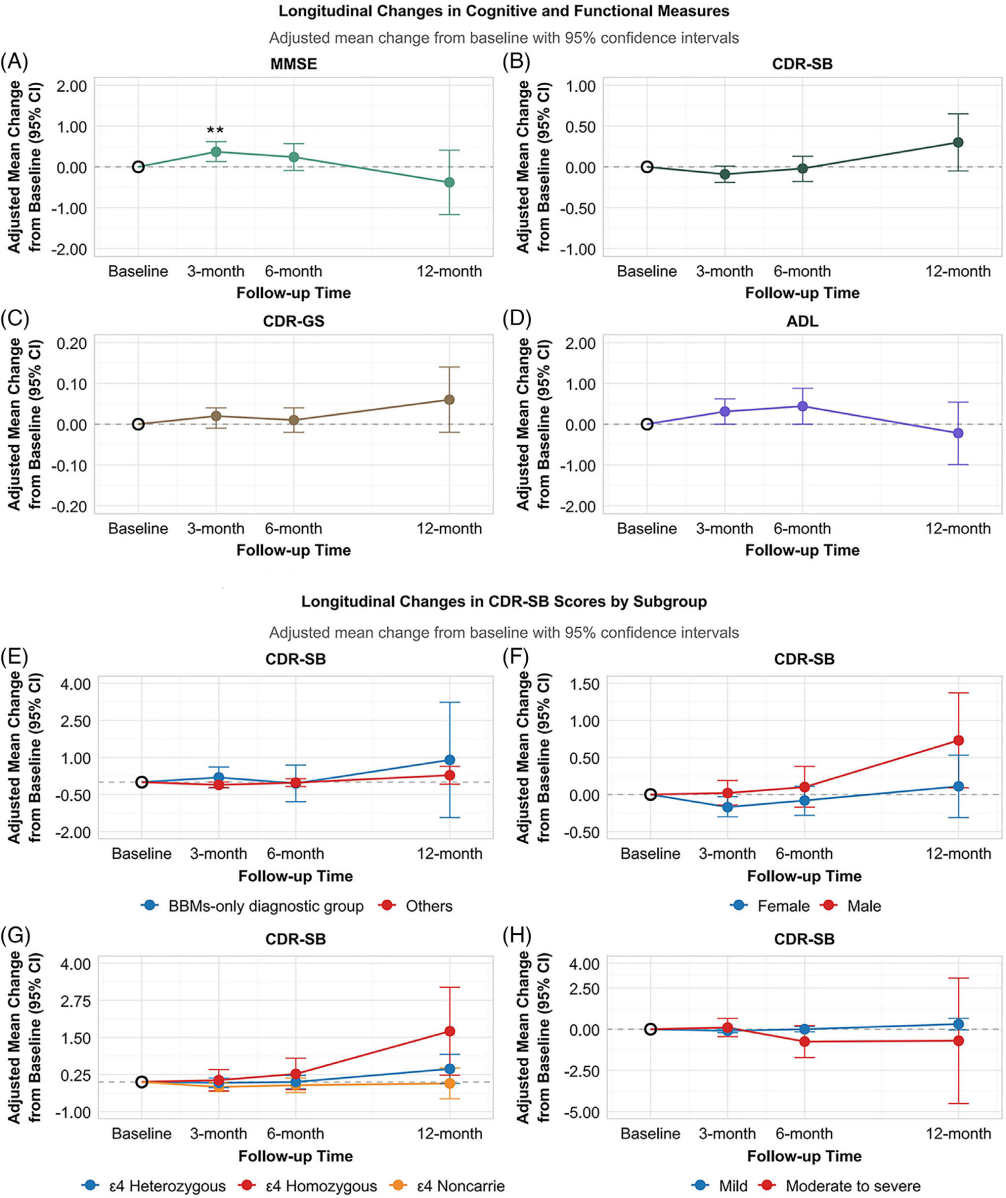
Longitudinal changes in cognitive scores from baseline to 12 months. (A–D) Adjusted mean change from baseline in MMSE (A), CDR‐SB (B), CDR‐GS(C), and ADL (D) scores over 3, 6, and 12 months of follow‐up. Panels E–H: subgroup analyses of adjusted mean CDR‐SB changes from baseline stratified by diagnostic group (E), sex (F), ApoE ε4 genotype (G), and disease severity (H) over 3, 6, and 12 months of follow‐up. All analyses were derived from an analysis of covariance (ANCOVA) model, which included baseline scores, age, and years of education as adjustment covariates to estimate adjusted least squares means. Error bars indicate 95% CIs. **indicates a statistically significant difference at P < 0.005; ns indicates no statistically significant difference at P ≥ 0.05. ADL, Activities of Daily Living; ApoE, apolipoprotein E; BBMs, blood‐based biomarkers; CDR‐GS, Clinical Dementia Rating–Global Score; CDR‐SB, Clinical Dementia Rating–Sum of Boxes; MMSE, Mini‐Mental State Examination

Using an ANCOVA model, we compared CDR‐SB, MMSE, CDR‐GS, and ADL scores at 3, 6, and 12 months (**Figure** **3**, eTable 9, eTable 10 in Supplement 1). For the primary outcome, CDR‐SB scores showed nonsignificant changes over 12 months. After adjustment for age, baseline cognitive function, and education, adjusted mean CDR‐SB changes were ‐0.09 (95% CI, ‐0.19 to 0.01; *P* = 0.082) at 3 months, ‐0.02 (95% CI, −0.18 to 0.13; *P* = 0.773) at 6 months, and 0.30 (95% CI, −0.05 to 0.65; *P* = 0.097) at 12 months. Among secondary cognitive measures, MMSE, CDR‐GS, and ADL scores showed no statistically significant change over 12 months. Across all subgroups, including sex, ApoE ε4 status, and baseline severity, 12‐month changes in CDR‐SB or MMSE did not significantly differ, with no clear evidence of differential cognitive trajectories over 12 months. We also compared longitudinal cognitive changes between patients diagnosed using BBMs alone and those diagnosed using other modalities. Across all time points, we did not observe statistically significant differences in CDR‐SB or MMSE changes between these diagnostic groups.

## 4 DISCUSSION

In this large, multicenter, real‐world observational cohort of 1,042 Chinese patients with AD, lecanemab demonstrated a manageable safety profile and no clear evidence of cognitive decline over 12 months. Our data suggest that high‐accuracy BBMs may offer a practical, non‐invasive approach to aid AD diagnosis and inform lecanemab treatment decisions, though these findings require further prospective validation.

The baseline characteristics of patients initiating lecanemab reflect the clinical epidemiological features of AD in real‐world Chinese settings. The cohort was slightly younger and had a higher proportion of female patients than the global Clarity AD trial^8^ and was aligned with previous real‐world studies in China (mean age 68 years, 67.6% female)^25^. This may indicate a stronger willingness among younger patients to pursue early treatment. The high proportion of women may be related to the higher incidence of AD in women,^3^, ^26^ Most patients had mild cognitive impairment or mild AD dementia, which is consistent with the approved indication for lecanemab in early AD.^4^ Notably, 6.14% of patients presented with moderate or severe dementia, suggesting that even individuals with progressive symptoms still actively seek treatment. This finding contrasts with the early‐stage selection bias in prior trials and highlights unmet treatment needs in broader clinical practice. ApoE ε4 carriers accounted for 49.52% of the cohort, lower than the ApoE ε4 carrier rate reported in the Clarity AD trial (68.9% globally and 72.6% in Asia).^8^ The ε4 allele is, however, less prevalent in Chinese sporadic AD populations^27^. Given that the ApoE ε4 allele is a core risk factor for ARIA,^28^ it is important to further delineate ARIA incidence and treatment response among Chinese ApoE ε4 carriers.

ARIA‐H may result from anti‐Aβ–mediated displacement of Aβ from parenchymal plaques to vessel walls, which can exacerbate pre‐existing cerebral amyloid angiopathy and lead to extravasation of blood products through damaged vessels.^29^, ^30^ Microhemorrhages often appear in areas where ARIA‐E resolves,^31^ suggesting overlapping mechanisms. Previous studies have defined patients who received at least four lecanemab infusions and one monitoring MRI as being at risk for ARIA and used this subgroup as the denominator when calculating ARIA incidence.^32^ However, eight patients in our cohort developed ARIA after fewer than four infusions. To account for this early‐onset ARIA and avoid underestimating incidence, we used the entire cohort of 1,042 patients as the denominator. In our cohort, the overall incidence of ARIA was 7.9% (ARIA‐E 2.9%, ARIA‐H 6.2%), which was lower than that reported in the global and Asian Clarity AD trials and in prior real‐world studies.^8^, ^25^, ^32^, ^33^, ^34^ Despite inclusion of patients with fewer than four infusions, ARIA incidence remained low, consistent with reports of greater ARIA tolerability in Asian populations. Our multivariable analysis identified baseline superficial siderosis, microhemorrhages, and ApoE ε4 homozygosity as strong independent predictors of all ARIA, consistent with previous studies.^28^, ^35^, ^36^ These findings underscore the importance of careful baseline MRI screening and early follow‐up to reduce ARIA risk. Multiple studies have demonstrated that a higher ApoE ε4 allele dose is associated with increased risk of ARIA.^29^, ^37^, ^38^, ^39^ The ApoE ε4 allele is associated with both higher total Aβ levels and greater vascular Aβ deposition.^40^, ^41^ Although some Asian studies did not detect this association,^25^, ^33^, ^42^ our findings in a large Chinese cohort support the relevance of ApoE ε4 stratification for ARIA risk. Although ApoE ε4 status, microhemorrhages, and superficial siderosis are recognized ARIA risk factors, many patients with these features still receive lecanemab in clinical practice. This may reflect the fact that most ARIA events are asymptomatic and occur early (within 6 months).^8^ Given the lack of association between vascular comorbidities or antithrombotic use and ARIA, these findings are consistent with the safe use of lecanemab in carefully selected patients^25^. IRRs occurred in 16.12% of patients, lower than reported in Clarity AD^8^ and within the range reported in Chinese real‐world studies^33^, ^34^. Most IRRs were mild‐to‐moderate, occurred during the first two infusions, and resolved spontaneously or with symptomatic treatment, supporting the feasibility of routine clinical monitoring without mandatory prophylactic medication. Treatment discontinuation occurred in 16.5% of patients, primarily due to financial burden and concern over adverse effects. In contrast to previous studies emphasizing ARIA‐related discontinuation,^32^ our findings highlight the economic barriers in China.

Clinical outcomes were evaluated using changes in CDR‐SB scores from baseline to 3, 6, and 12 months, as in the Clarity AD trial^8^. Mean CDR‐SB scores did not significantly change over 12 months, consistent with previous studies.^8^, ^25^, ^43^ The absence of statistically significant differences in cognitive and functional measures across sex, ApoE ε4 status, and disease severity indicates that no differential patterns were observed across these subgroups. It must be emphasized that this was an observational cohort study without randomized controls; therefore, a causal relationship between lecanemab treatment and the observed cognitive outcomes should not be interpreted as evidence of treatment effect. The Alzheimer's Disease Neuroimaging Initiative cohort reported an annual CDR–SB progression rate of approximately 0.5 points per year in patients with late mild cognitive impairment and 1.4 points per year in patients with mild AD.^44^ In our single‐arm cohort, the adjusted mean change in CDR‐SB from baseline to month 12 was 0.30 (95% CI: −0.05 to 0.65), a rate lower than the reported natural history progression. We explicitly acknowledge, however, that this cross‐study comparison is exploratory and hypothesis‐generating only It does not replace a concurrent control arm, and no causal inference regarding lecanemab efficacy can be drawn from this comparison. Despite this limitation, in clinical practice where patients with moderate‑to‑severe Alzheimer's disease often have high unmet needs and strong treatment motivation, these findings offer timely real‑world evidence to support individualized clinical decision‑making. From a practical standpoint, the absence of statistically significant differences across subgroups suggests that factors such as sex and ApoE ε4 status did not differentially influence treatment response in this cohort. Consequently, they may not be primary determinants for excluding patients from lecanemab therapy.

Patients diagnosed via BBMs (5.28% of our cohort) showed cognitive trajectories comparable to those diagnosed using PET or CSF, providing preliminary evidence for the potential clinical utility of convenient and cost‐effective BBMs for treatment selection. BBMs for AD are increasingly used in clinical practice to support diagnosis.^12^, ^45^, ^46^ Compared with traditional modalities such as Aβ‐PET and CSF analysis, BBMs offer a more accessible and lower‐cost alternative for confirming amyloid pathology. Their scalability is particularly valuable given the rising demand for biomarker testing associated with disease‐modifying therapies. To our knowledge, this is the first real‐world study to report that high‐accuracy BBMs may serve as standalone diagnostic tools to guide lecanemab treatment decisions in AD.

In terms of limitations, although participant characteristics at follow‐up were relatively stable compared with baseline, substantial attrition during follow‐up may introduce selection bias, as patients who discontinued treatment or were lost to follow‐up may differ systematically from those who remained in the cohort. As a multicenter study, variability in MRI monitoring protocols and ARIA detection criteria across sites may exist. Finally, given the small BBMs sample size and the lack of post‐treatment longitudinal BBMs data, which limits assessment of their utility for monitoring treatment response, these findings reflect preliminary real‐world feasibility and require validation in larger cohorts.

In summary, our findings provide longer‐term, risk‐stratified evidence that lecanemab appears safe and feasible in routine Chinese clinical practice, with no clear evidence of cognitive decline. As the first real‐world study to include patients diagnosed using BBMs for efficacy assessment, this work provides preliminary evidence supporting the potential diagnostic utility of these non‐invasive assays, which requires further prospective validation. Collectively, these results provide preliminary real‐world evidence to inform lecanemab treatment decisions and long‐term monitoring strategies for Chinese patients with AD.

## AUTHOR CONTRIBUTIONS

All authors contributed to the work, approved the final manuscript, and are accountable for their contributions. Drs Yong Ji, Xiaochun Chen, Guoping Peng, Qin Chen, Min Fang, Huidong Tang, Huayan Liu, Jun Liu, and Jiewen Zhang had full access to all the data and take responsibility for the integrity and accuracy of the data analysis. Drs. Hao Wu, Qin Chen, Min Fang, Huidong Tang, Huayan Liu, Jun Liu, Jiewen Zhang, Lijun Chi, Shuai Liu and Jiawei Xin share co‐first authorship. Study concept and design: Yong Ji, Xiaochun Chen, Hao Wu, Shuai Liu, Jiawei Xin and Min Fang. Acquisition, analysis, or interpretation of data: All authors. Drafting of the manuscript: Hao Wu, Yong Ji and Shuai Liu. Critical revision of the manuscript for important intellectual content: All authors. Statistical analysis: Hao Wu and Yong Ji. Obtained funding: Yong Ji. Administrative, technical, or material support: All authors. Study supervision: Yong Ji, Xiaochun Chen, Guoping Peng, Qin Chen, Min Fang, Huidong Tang, Huayan Liu, Jun Liu, Jiewen Zhang and Lijun Chi.

## CONFLICT OF INTEREST STATEMENT

The authors declare no actual or potential financial or other conflicts of interest related to this manuscript. Author disclosures are available in the Supporting Information. Author disclosures are available in the Supporting Information.

## ETHICS APPROVAL AND CONSENT TO PARTICIPATE

The study protocol was approved by the institutional review board of Tianjin Huanhu Hospital, which acted as the central ethics committee (ID: 2025‐305). Informed consent was obtained from all participants or their legal representatives.

## CONSENT STATEMENT

All authors approved its publication

## Supporting information



Supporting Information

Supporting Information

## Data Availability

Aggregated or de‐identified data are available upon reasonable request and with approval from the institutional ethics committee. Requests should be directed to the corresponding author.
