# Supplementary material for: Lecanemab use in Chinese patients with Alzheimer's disease: a 12‐month multicenter real‐world study
Source: Alzheimers Dement. 2026 Aug 6;22(8):e71702. doi: 10.1002/alz.71702 (PMC13444352; doi:10.1002/alz.71702)
Supplement: Supplementary file 2 [file ALZ-22-e71702-s002.docx]

**Supplemental Materials**

**Lecanemab Use in Chinese Patients With Alzheimer Disease: A 12-Month Multicenter Real-World Study**

**Contents**

**Supplemental Tables**

1. eTable 1. Baseline Clinical and Demographic Characteristics of Patients Stratified by Diagnostic Biomarker Type
2. eTable 2. Baseline Characteristics of Patients With and Without Amyloid-Related Imaging Abnormalities (ARIA)
3. eTable 3. Baseline Characteristics of All Amyloid-Related Imaging Abnormalities (ARIA) Subtypes and Associated Factors
4. eTable 4. Occurrence and Severity of Amyloid-Related Imaging Abnormalities (ARIA) Subgroups
5. eTable 5. Incidence and Severity of Infusion-Related Reactions (IRRs) by Infusion Number
6. eTable 6. Baseline Characteristics of Discontinuation Treatment Group
7. eTable 7. Reasons for Discontinuation of Lecanemab Treatment by Infusion Number
8. eTable 8. Follow-Up Demographic and Cognitive Characteristics
9. eTable 9. Changes in Cognitive Assessment Scores From Baseline to 12-Month Follow-Up
10. eTable 10. Follow-Up Subgroup analyses of CDR-SB and MMSE changes by diagnostic group, sex, disease severity and Apolipoprotein E (ApoE) ε4 genotype

**Supplemental Figures**

1. eFigure 1. The flow chart of the study
2. eFigure 2. Reasons for Treatment Discontinuation

**eTable 1. Baseline Clinical and Demographic Characteristics of Patients Stratified by Diagnostic Biomarker Type**

| **Characteristic** | **BBMs-only**  **diagnostic group** | **Other**  **diagnostic group** | ***P* value** |
| --- | --- | --- | --- |
| Participants, No. | 55 | 987 | - |
| Age, mean (SD), y | 71.05 (6.76) | 68.81 (8.68) | 0.081 |
| Sex, No. (%) |  |  | 0.975 |
| Male | 20 (36.36) | 361 (36.58) |  |
| Female | 35 (63.64) | 626 (63.42) |  |
| Education, mean (SD), y | 11.11 (3.65) | 11.36 (4.04) | 0.351 |
| Weight, mean (SD), kg | 60.62 (11.24) | 59.92 (9.87) | 0.525 |
| MMSE score, mean (SD) | 18.25 (5.08) | 20.03 (4.95) | 0.006 |
| MoCA score, mean (SD) | 13.94 (4.90) | 15.62 (5.27) | 0.035 |
| CDR-GS, No. (%) |  |  | 0.025 |
| 0.5 | 30 (54.55) | 579 (58.66) |  |
| 1 | 18 (32.73) | 244 (24.72) |  |
| 2 | 0 | 55 (5.57) |  |
| 3 | 2 (3.64) | 7 (0.71) |  |
| Unknown | 5 (9.09) | 102 (10.33) |  |
| CDR-SB score, mean (SD) | 4.23 (3.79) | 3.79 (2.74) | 0.679 |
| ADL score, mean (SD) | 25.91 (7.67) | 25.29 (6.68) | 0.808 |
| ApoE Status, No. (%) |  |  | 0.234 |
| ε2/ε3 | 3 (5.45) | 70 (7.09) |  |
| ε3/ε3 | 15 (27.27) | 379 (38.40) |  |
| ε2/ε4 | 2 (3.64) | 43 (4.36) |  |
| ε3/ε4 | 28 (50.91) | 350 (35.46) |  |
| ε4/ε4 | 6 (10.91) | 87 (8.81) |  |
| Unknown | 1 (1.82) | 58 (5.88) |  |

Abbreviations: ADL, Activities of Daily Living; ApoE, apolipoprotein E; BBMs, blood-based biomarkers; CDR-GS, Clinical Dementia Rating-Global Scale; CDR-SB, Clinical Dementia Rating-Sum of Boxes; MMSE, Mini-Mental State Examination; MoCA, Montreal Cognitive Assessment.

**eTable 2. Baseline Characteristics of Patients With and Without Amyloid-Related Imaging Abnormalities (ARIA)**

| **Characteristic** | **Total** | **No ARIA** | **Any ARIA** | ***P* value*^a^*** | **ARIA-E** | ***P* value*^a^*** | **ARIA-H** | ***P* value*^a^*** |
| --- | --- | --- | --- | --- | --- | --- | --- | --- |
| Participants, No. (%) | 1042 (100) | 960 (92.13) | 82 (7.87) | - | 30 (2.88) | - | 65 (6.24) | - |
| Age, mean (SD), y | 68.93 (8.60) | 68.82 (8.49) | 70.27 (9.79) | 0.100 | 67.10 (10.54) | 0.335 | 71.54 (9.44) | 0.011 |
| Sex, No. (%) |  |  |  | 0.997 |  | 0.449 |  | 0.49 |
| Male | 381 (36.56) | 351 (36.56) | 30 (36.59) |  | 13 (43.33) |  | 21 (32.31) |  |
| Female | 661 (63.44) | 609 (63.44) | 52 (63.41) |  | 17 (56.67) |  | 44 (67.69) |  |
| Education, mean (SD), y | 11.35 (4.02) | 11.32 (4.07) | 11.64 (3.47) | 0.557 | 11.78 (3.58) | 0.573 | 11.62 (3.51) | 0.624 |
| Height, mean (SD), cm | 163.60 (8.38) | 163.61 (8.44) | 163.48 (7.63) | 0.765 | 165.68 (8.71) | 0.356 | 163.05 (7.49) | 0.504 |
| Weight, mean (SD), kg | 59.95 (9.95) | 60.13 (9.83) | 57.89 (11.06) | 0.059 | 60.51 (10.57) | 0.944 | 56.55 (10.89) | 0.007 |
| MMSE score, mean (SD) | 19.94 (4.97) | 19.88 (5.01) | 20.65 (4.48) | 0.179 | 21.00 (4.16) | 0.236 | 20.42 (4.67) | 0.412 |
| MoCA score, mean (SD) | 15.51 (5.26) | 15.53 (5.33) | 15.33 (4.32) | 0.707 | 14.96 (4.83) | 0.652 | 15.24 (4.19) | 0.578 |
| CDR-GS, No. (%) |  |  |  | 0.423 |  | 0.931 |  | 0.689 |
| 0.5 | 609 (58.45) | 556 (57.92) | 53 (64.63) |  | 20 (66.67) |  | 42 (64.62) |  |
| 1 | 262 (25.14) | 235 (24.48) | 27 (32.93) |  | 9 (30.00) |  | 21 (32.31) |  |
| 2 | 55 (5.28) | 53 (5.52) | 2 (2.44) |  | 1 (3.33) |  | 2 (3.08) |  |
| 3 | 9 (0.86) | 9 (0.94) | 0 |  | 0 |  | 0 |  |
| Unknown | 107 (10.27) | 107 (11.15) | 0 |  | 0 |  | 0 |  |
| CDR-SB score, mean (SD) | 3.82 (2.81) | 3.81 (2.87) | 3.91 (2.06) | 0.136 | 3.33 (1.95) | 0.705 | 4.10 (2.08) | 0.052 |
| ADL score, mean (SD) | 25.32 (6.73) | 25.38 (6.80) | 24.70 (5.95) | 0.458 | 24.32 (5.00) | 0.563 | 25.07 (6.18) | 0.92 |
| ApoE ε4 Status, No. (%) |  |  |  | <0.001 |  | 0.088 |  | <0.001 |
| ε4 Noncarrier | 467 (44.82) | 440 (45.83) | 27 (32.93) |  | 12 (40.00) |  | 19 (29.23) |  |
| ε4 Heterozygous | 423 (40.60) | 385 (40.10) | 38 (46.34) |  | 12 (40.00) |  | 32 (49.23) |  |
| ε4 Homozygous | 93 (8.93) | 76 (7.92) | 17 (20.73) |  | 6 (20.00) |  | 14 (21.54) |  |
| Unknown | 59 (5.66) | 59 (6.15) | 0 |  | 0 |  | 0 |  |
| Comorbidities, No. (%) |  |  |  |  |  |  |  |  |
| Hypertension | 291 (27.93) | 263 (27.40) | 28 (34.15) | 0.191 | 7 (23.33) | 0.623 | 23 (35.38) | 0.165 |
| Diabetes | 170 (16.31) | 162 (16.88) | 8 (9.76) | 0.094 | 2 (6.67) | 0.209 | 8 (12.31) | 0.338 |
| Hyperlipidemia | 172 (16.51) | 156 (16.25) | 16 (19.51) | 0.445 | 2 (6.67) | 0.208 | 14 (21.54) | 0.267 |
| Coronary heart disease | 83 (7.97) | 76 (7.92) | 7 (8.54) | 0.842 | 1 (3.33) | 0.724 | 6 (9.23) | 0.705 |
| Atrial fibrillation | 22 (2.11) | 21 (2.19) | 1 (1.22) | 1 | 0 | 1 | 1 (1.54) | 1 |
| Cerebral infarction | 48 (4.61) | 42 (4.38) | 6 (7.32) | 0.263 | 1 (3.33) | 1 | 5 (7.69) | 0.214 |
| Medication use, No. (%) |  |  |  |  |  |  |  |  |
| Cholinesterase inhibitor | 618 (59.31) | 574 (59.79) | 44 (53.66) | 0.278 | 18 (60.00) | 0.982 | 34 (52.31) | 0.235 |
| Memantine | 263 (25.24) | 241 (25.10) | 22 (26.83) | 0.73 | 9 (30.00) | 0.543 | 17 (26.15) | 0.85 |
| Anticoagulant | 8 (0.77) | 7 (0.73) | 1 (1.22) | 0.482 | 0 | 1 | 1 (1.54) | 0.409 |
| Antiplatelet | 83 (7.97) | 74 (7.71) | 9 (10.98) | 0.294 | 3 (10.00) | 0.502 | 6 (9.23) | 0.658 |
| Antihypertensive | 243 (23.32) | 219 (22.81) | 24 (29.27) | 0.185 | 7 (23.33) | 0.947 | 20 (30.77) | 0.142 |
| Antidiabetic drugs | 148 (14.20) | 140 (14.58) | 8 (9.76) | 0.229 | 2 (6.67) | 0.296 | 8 (12.31) | 0.613 |
| Microhemorrhages, No. (%) |  |  |  | <0.001 |  | 0.006 |  | <0.001 |
| 0 | 856 (82.15) | 804 (83.75) | 52 (63.41) |  | 23 (76.67) |  | 38 (58.46) |  |
| 1–4 | 173 (16.60) | 148 (15.42) | 25 (30.49) |  | 4 (13.33) |  | 22 (33.85) |  |
| ≥5 | 13 (1.25) | 8 (0.83) | 5 (6.10) |  | 3 (10.00) |  | 5 (7.69) |  |
| Superficial siderosis, No. (%) |  |  |  | <0.001 |  | <0.001 |  | <0.001 |
| 0 | 1024 (98.27) | 952 (99.17) | 72 (87.80) |  | 22 (73.33) |  | 58 (89.23) |  |
| 1 | 18 (1.73) | 8 (0.83) | 10 (12.20) |  | 8 (26.67) |  | 7 (10.77) |  |
| Fazekas score, No. (%) |  |  |  | 0.178 |  | 0.547 |  | 0.079 |
| 0 | 88 (8.45) | 79 (8.23) | 9 (10.98) |  | 2 (6.67) |  | 8 (12.31) |  |
| 1 | 447 (42.90) | 420 (43.75) | 27 (32.93) |  | 13 (43.33) |  | 18 (27.69) |  |
| 2 | 156 (14.97) | 144 (15.00) | 12 (14.63) |  | 2 (6.67) |  | 10 (15.38) |  |
| 3 | 28 (2.69) | 24 (2.50) | 4 (4.88) |  | 1 (3.33) |  | 3 (4.62) |  |
| Unknown | 323 (31.00) | 293 (30.52) | 30 (36.59) |  | 12 (40.00) |  | 26 (40.00) |  |

Abbreviations: ADL, Activities of Daily Living; ApoE, apolipoprotein E; ARIA, amyloid-related imaging abnormality; ARIA-E, amyloid-related imaging abnormality-edema/effusion; ARIA-H, amyloid-related imaging abnormality-hemorrhage/hemosiderin deposition; CDR-GS, Clinical Dementia Rating-Global Scale; CDR-SB, Clinical Dementia Rating-Sum of Boxes; MMSE, Mini-Mental State Examination; MoCA, Montreal Cognitive Assessment;.

^a^ All *P* values correspond to comparisons between the indicated group (Any ARIA, ARIA-E, ARIA-H) and the No ARIA group

**eTable 3. Baseline Characteristics of All Amyloid-Related Imaging Abnormalities (ARIA) Subtypes and Associated Factors**

| **Characteristic** | **Asymptomatic ARIA-E** | **Symptomatic ARIA-E** | **Isolated ARIA-E** | **ARIA-E with ARIA-H** | **Asymptomatic ARIA-H** | **Symptomatic ARIA-H** | **Isolated ARIA-H** |
| --- | --- | --- | --- | --- | --- | --- | --- |
| Participants, No. (%) | 26 (2.50) | 4 (0.38) | 17 (1.63) | 13 (1.25) | 58 (5.57) | 7 (0.67) | 52 (4.99) |
| Age, mean (SD), y | 66.23 (10.80) | 72.75 (7.14) | 65.41 (9.83) | 69.31 (11.40) | 71.00 (9.41) | 76.00 (9.17)* | 72.10 (8.93)* |
| Sex, No. (%) |  |  |  |  |  |  |  |
| Male | 11 (42.31) | 2 (50.00) | 9 (52.94) | 4 (30.77) | 19 (32.76) | 2 (28.57) | 17 (32.69) |
| Female | 15 (57.69) | 2 (50.00) | 8 (47.06) | 9 (69.23) | 39 (67.24) | 5 (71.43) | 35 (67.31) |
| Education, mean (SD), y | 11.88 (3.70) | 11.13 (3.01) | 11.71 (3.44) | 11.88 (3.90) | 11.61 (3.40) | 11.64 (4.63) | 11.55 (3.44) |
| Weight, mean (SD), kg | 60.36 (10.55) | 61.53 (12.27) | 62.93 (10.53) | 57.35 (10.15) | 56.62 (11.34)* | 56.04 (6.75) | 56.35 (11.16)* |
| MMSE score, mean (SD) | 20.73 (4.07) | 22.75 (4.99) | 21.53 (3.62) | 20.31 (4.84) | 20.34 (4.84) | 21.00 (3.21) | 20.44 (4.68) |
| MoCA score, mean (SD) | 15.05 (4.52) | 14.50 (6.95) | 15.67 (4.96) | 14.18 (4.79) | 15.50 (4.13) | 13.20 (4.55) | 15.59 (3.99) |
| CDR-GS, No. (%) |  |  |  |  |  |  |  |
| 0.5 | 18 (69.23) | 2 (50.00) | 11 (64.71) | 9 (69.23) | 38 (65.52) | 4 (57.14) | 33 (63.46) |
| 1 | 8 (30.77) | 1 (25.00) | 6 (35.29) | 3 (23.08) | 19 (32.76) | 2 (28.57) | 18 (34.62) |
| 2 | 0 | 1 (25.00) | 0 | 1 (7.69) | 1 (1.72) | 1 (14.29) | 1 (1.92) |
| 3 | 0 | 0 | 0 | 0 | 0 | 0 | 0 |
| Unknown | 0 | 0 | 0 | 0 | 0 | 0 | 0 |
| CDR-SB score, mean (SD) | 3.24 (1.77) | 3.75 (2.99) | 3.18 (1.90) | 3.56 (2.13) | 3.92 (1.94) | 5.36 (2.70) | 4.21 (2.08)* |
| ADL score, mean (SD) | 24.14 (5.22) | 25.25 (4.11) | 23.21 (4.85) | 25.73 (5.04) | 24.42 (5.15) | 30.50 (10.99) | 24.91 (6.47) |
| ApoE ε4 Status, No. (%) |  |  |  |  | * |  | * |
| ε4 Noncarrier | 12 (46.15) | 0 | 8 (47.06) | 4 (30.77) | 18 (31.03) | 1 (14.29) | 15 (28.85) |
| ε4 Heterozygous | 9 (34.62) | 3 (75.00) | 6 (35.29) | 6 (46.15) | 28 (48.28) | 4 (57.14) | 26 (50.00) |
| ε4 Homozygous | 5 (19.23) | 1 (25.00) | 3 (17.65) | 3 (23.08) | 12 (20.69) | 2 (28.57) | 11 (21.15) |
| Unknown | 0 | 0 | 0 | 0 | 0 | 0 | 0 |
| Comorbidities, No. (%) |  |  |  |  |  |  |  |
| Hypertension | 7 (26.92) | 0 | 5 (29.41) | 2 (15.38) | 19 (32.76) | 4 (57.14) | 21 (40.38)* |
| Diabetes | 1 (3.85) | 1 (25.00) | 0 | 2 (15.38) | 6 (10.34) | 2 (28.57) | 6 (11.54) |
| Hyperlipidemia | 2 (7.69) | 0 | 2 (11.76) | 0 | 12 (20.69) | 2 (28.57) | 14 (26.92)* |
| Coronary heart disease | 1 (3.85) | 0 | 1 (5.88) | 0 | 4 (6.90) | 2 (28.57) | 6 (11.54) |
| Atrial fibrillation | 0 | 0 | 0 | 0 | 1 (1.72) | 0 | 1 (1.92) |
| Cerebral infraction | 1 (3.85) | 0 | 1 (5.88) | 0 | 4 (6.90) | 1 (14.29) | 5 (9.62) |
| Medication use, No. (%) |  |  |  |  |  |  |  |
| Anticholinesterase | 14 (53.85) | 4 (100.00) | 10 (58.82) | 8 (61.54) | 30 (51.72) | 4 (57.14) | 26 (50.00) |
| Memantine | 6 (23.08) | 3 (75.00) | 5 (29.41) | 4 (30.77) | 14 (24.14) | 3 (42.86) | 13 (25.00) |
| Anticoagulant | 0 | 0 | 0 | 0 | 1 (1.72) | 0 | 1 (1.92) |
| Antiplatelet | 2 (7.69) | 1 (25.00) | 3 (17.65) | 0 | 4 (6.90) | 2 (28.57) | 6 (11.54) |
| Antihypertensive | 7 (26.92) | 0 | 4 (23.53) | 3 (23.08) | 16 (27.59) | 4 (57.14) | 17 (32.69) |
| Antidiabetic drugs | 1 (3.85) | 1 (25.00) | 0 | 2 (15.38) | 6 (10.34) | 2 (28.57) | 6 (11.54) |
| Microhemorrhages, No. (%) |  | * |  | * | * | * | * |
| 0 | 22 (84.62) | 1 (25.00) | 14 (82.35) | 9 (69.23) | 34 (58.62) | 4 (57.14) | 29 (55.77) |
| 1~4 | 3 (11.54) | 1 (25.00) | 3 (17.65) | 1 (7.69) | 21 (36.21) | 1 (14.29) | 21 (40.38) |
| >4 | 1 (3.85) | 2 (50.00) | 0 | 3 (23.08) | 3 (5.17) | 2 (28.57) | 2 (3.85) |
| Superficial siderosis,No. (%) | * | * | * | * | * |  |  |
| 0 | 19 (73.08) | 3 (75.00) | 14 (82.35) | 8 (61.54) | 52 (89.66) | 6 (85.71) | 50 (96.15) |
| 1 | 7 (26.92) | 1 (25.00) | 3 (17.65) | 5 (38.46) | 6 (10.34) | 1 (14.29) | 2 (3.85) |
| Fazekas score, No. (%) |  |  |  |  |  |  | * |
| 0 | 2 (7.69) | 0 | 1 (5.88) | 1 (7.69) | 6 (10.34) | 2 (28.57) | 7 (13.46) |
| 1 | 10 (38.46) | 3 (75.00) | 9 (52.94) | 4 (30.77) | 17 (29.31) | 1 (14.29) | 14 (26.92) |
| 2 | 2 (7.69) | 0 | 2 (11.76) | 0 | 8 (13.79) | 2 (28.57) | 10 (19.23) |
| 3 | 1 (3.85) | 0 | 1 (5.88) | 0 | 3 (5.17) | 0 | 3 (5.77) |
| Unknown | 11 (42.31) | 1 (25.00) | 4 (23.53) | 8 (61.54) | 24 (41.38) | 2 (28.57) | 18 (34.62) |

Abbreviations: ADL, Activities of Daily Living; ApoE, apolipoprotein E; CDR-GS, Clinical Dementia Rating-Global Scale; CDR-SB, Clinical Dementia Rating-Sum of Boxes; HAMA, Hamilton Anxiety Rating Scale; HAMD, Hamilton Depression Rating Scale; MMSE, Mini-Mental State Examination; MoCA, Montreal Cognitive Assessment; NPI, Neuropsychiatric Inventory.

*indicates a statistically significant difference at *P* < 0.05 for comparisons between the indicated ARIA subgroup and the No ARIA group.

**eTable 4. Occurrence and Severity of Amyloid-Related Imaging Abnormalities (ARIA) Subgroups**

| **Characteristic** | **Age (years)** | **ApoEε4 Status** | | |  | **CDR-GS** | | | |  | **Severity** | | |
| --- | --- | --- | --- | --- | --- | --- | --- | --- | --- | --- | --- | --- | --- |
|  |  | **Noncarrier** | **Heterozygous** | **Homozygous** |  | **0.5** | **1** | **2** | **3** |  | **Mild** | **Moderate** | **Severe** |
| No ARIA, n=960 | 68.82 (8.49) | 440 (45.83) | 385 (40.10) | 76 (7.92) |  | 556 (57.92) | 235 (24.48) | 53 (5.52) | 9 (0.94) |  | - | - | - |
| Any ARIA, n=82 | 70.27 (9.79) | 27 (32.93) | 38 (46.34) | 17 (20.73) |  | 53 (64.63) | 27 (32.93) | 2 (2.44) | 0 |  | 60 (73.17) | 14 (17.07) | 8 (9.76) |
| ARIA-E, n=30 | 67.10 (10.54) | 12 (40.00) | 12 (40.00) | 6 (20.00) |  | 20 (66.67) | 9 (30.00) | 1 (3.33) | 0 |  | 20 (66.67) | 10 (33.33) | 0 |
| Asymptomatic  ARIA-E,n=26 | 66.23 (10.80) | 12 (46.15) | 9 (34.62) | 5 (19.23) |  | 18 (69.23) | 8 (30.77) | 0 | 0 |  | 19 (73.08) | 7 (26.92) | 0 |
| Symptomatic  ARIA-E, n=4 | 72.75 (7.14) | 0 | 3 (75.00) | 1 (25.00) |  | 2 (50.00) | 1 (25.00) | 1 (25.00) | 0 |  | 1 (25.00) | 3 (75.00) | 0 |
| ARIA-H, n=65 | 71.54 (9.44) | 19 (29.23) | 32 (49.23) | 14 (21.54) |  | 42 (64.62) | 21 (32.31) | 2 (3.08) | 0 |  | 50 (76.92) | 7 (10.77) | 8 (12.31) |
| Asymptomatic  ARIA-H,n=58 | 71.00 (9.41) | 18 (31.03) | 28 (48.28) | 12 (20.69) |  | 38 (65.52) | 19 (32.76) | 1 (1.72) | 0 |  | 49 (84.48) | 4 (6.90) | 5 (8.62) |
| Symptomatic  ARIA-H, n=7 | 76.00 (9.17) | 1 (14.29) | 4 (57.14) | 2 (28.57) |  | 4 (57.14) | 2 (28.57) | 1 (14.29) | 0 |  | 1 (14.29) | 3 (42.86) | 3 (42.86) |
| ARIA-E with  ARIA-H, n=13 | 69.31 (11.40) | 4 (30.77) | 6 (46.15) | 3 (23.08) |  | 9 (69.23) | 3 (23.08) | 1 (7.69) | 0 |  | 8 (61.54) | 4 (30.77) | 1 (7.69) |

Abbreviations: ApoE, apolipoprotein E; ARIA, amyloid-related imaging abnormality; ARIA-E, amyloid-related imaging abnormality–edema; ARIA-H, amyloid-related imaging abnormality–hemorrhage; CDR-GS, Clinical Dementia Rating-Global Scale.

Values are presented as mean (SD) or No. (%).

**eTable 5. Incidence and Severity of Infusion-Related Reactions (IRRs) by Infusion Number**

| **Infusion Number** | **Total IRRs, n (%)** | **Severity Grade** | **IRRs with Fever/Chills, n (%)** | **IRRs without Fever/Chills, n (%)** |
| --- | --- | --- | --- | --- |
| All Infusions | 168 (16.1) | - | - | - |
| 1st | 146 (14.01) | Mild | 95 (9.12) | 35 (3.36) |
|  |  | Moderate | 11 (1.06) | 2 (0.19) |
|  |  | Severe | 1 (0.10) | 2 (0.19) |
| 2nd | 8 (0.77) | Mild | 3 (0.29) | 5 (0.48) |
|  |  | Moderate | 0 (0.00) | 0 (0.00) |
|  |  | Severe | 0 (0.00) | 0 (0.00) |
| 3rd | 3 (0.29) | Mild | 1 (0.10) | 2 (0.19) |
|  |  | Moderate | 0 (0.00) | 0 (0.00) |
|  |  | Severe | 0 (0.00) | 0 (0.00) |
| 4th | 3 (0.29) | Mild | 1 (0.10) | 0 (0.00) |
|  |  | Moderate | 0 (0.00) | 2 (0.19) |
|  |  | Severe | 0 (0.00) | 0 (0.00) |
| 5th | 1 (0.10) | Mild | 0 (0.00) | 1 (0.10) |
|  |  | Moderate | 0 (0.00) | 0 (0.00) |
|  |  | Severe | 0 (0.00) | 0 (0.00) |
| 10th | 2 (0.19) | Mild | 0 (0.00) | 0 (0.00) |
|  |  | Moderate | 0 (0.00) | 2 (0.19) |
|  |  | Severe | 0 (0.00) | 0 (0.00) |
| 15th | 1 (0.10) | Mild | 0 (0.00) | 1 (0.10) |
|  |  | Moderate | 0 (0.00) | 0 (0.00) |
|  |  | Severe | 0 (0.00) | 0 (0.00) |
| 17th | 1 (0.10) | Mild | 0 (0.00) | 1 (0.10) |
|  |  | Moderate | 0 (0.00) | 0 (0.00) |
|  |  | Severe | 0 (0.00) | 0 (0.00) |
| 18th | 1 (0.10) | Mild | 0 (0.00) | 1 (0.10) |
|  |  | Moderate | 0 (0.00) | 0 (0.00) |
|  |  | Severe | 0 (0.00) | 0 (0.00) |
| 25th | 1 (0.10) | Mild | 0 (0.00) | 1 (0.10) |
|  |  | Moderate | 0 (0.00) | 0 (0.00) |
|  |  | Severe | 0 (0.00) | 0 (0.00) |
| 36th | 1 (0.10) | Mild | 0 (0.00) | 1 (0.10) |
|  |  | Moderate | 0 (0.00) | 0 (0.00) |
|  |  | Severe | 0 (0.00) | 0 (0.00) |

Abbreviations: IRRs, Infusion-related reactions

**eTable 6. Baseline Characteristics of Discontinuation Treatment Group**

| **Characteristic** | **Total** | **Discontinuation treatment group** | **Non-Discontinuation treatment group** | ***P* value** |
| --- | --- | --- | --- | --- |
| Participants, No. | 1042 | 172 | 870 |  |
| Age, mean (SD), y | 68.93 (8.60) | 69.83 (8.98) | 68.75 (8.52) | 0.176 |
| Sex, No. (%) |  |  |  | 0.290 |
| Male | 381 (36.56) | 69 (40.12) | 312 (35.86) |  |
| Female | 661 (63.44) | 103 (59.88) | 558 (64.14) |  |
| Education, mean (SD), y | 11.35 (4.02) | 11.27 (4.12) | 11.36 (4.00) | 0.636 |
| Weight, mean (SD), kg | 59.95 (9.95) | 59.22 (11.12) | 60.08 (9.73) | 0.258 |
| MMSE score, mean (SD) | 19.94 (4.97) | 19.34 (5.37) | 20.05 (4.88) | 0.122 |
| MoCA score, mean (SD) | 15.51 (5.26) | 15.21 (5.43) | 15.58 (5.23) | 0.726 |
| CDR-GS, No. (%) |  |  |  | 0.004 |
| 0.5 | 609 (58.45) | 87 (50.58) | 522 (60.00) |  |
| 1 | 262 (25.14) | 59 (34.30) | 203 (23.33) |  |
| 2 | 55 (5.28) | 15 (8.72) | 40 (4.60) |  |
| 3 | 9 (0.86) | 2 (1.16) | 7 (0.80) |  |
| Unknow | 107 (10.27) | 9 (5.23) | 98 (11.26) |  |
| CDR-SB score, mean (SD) | 3.82 (2.81) | 4.80 (3.14) | 3.64 (2.71) | <0.001 |
| ADL score, mean (SD) | 23 (20,28) | 24 (20,31) | 23 (20,28) | 0.005 |
| Total NPI score, mean (SD) | 2 (0,8) | 1 (0,8) | 2 (0,8) | 0.261 |
| ApoE Status, No. (%) |  |  |  | 0.229 |
| ε2/ε3 | 73 (7.01) | 16 (9.30) | 57 (6.55) |  |
| ε3/ε3 | 394 (37.81) | 57 (33.14) | 337 (38.74) |  |
| ε2/ε4 | 45 (4.32) | 12 (6.98) | 33 (3.79) |  |
| ε3/ε4 | 378 (36.28) | 59 (34.30) | 319 (36.67) |  |
| ε4/ε4 | 93 (8.93) | 18 (10.47) | 75 (8.62) |  |
| Unknown | 59 (5.66) | 10 (5.81) | 49 (5.63) |  |

Abbreviations: ADL, Activities of Daily Living; ApoE, apolipoprotein E; CDR-GS, Clinical Dementia Rating-Global Scale; CDR-SB, Clinical Dementia Rating-Sum of Boxes; MMSE, Mini-Mental State Examination; MoCA, Montreal Cognitive Assessment; NPI, Neuropsychiatric Inventory.

^a^ Values are presented as mean (SD) or No. (%).

**eTable 7. Reasons for Discontinuation of Lecanemab Treatment by Infusion Number**

| **Infusion Number** | **Total Discontinuations, n (%)** | **Reasons for Discontinuation, n (%)** |
| --- | --- | --- |
| All | 172 (100.00) | - |
| 1st | 13 (7.56%) | Symptomatic ARIA (1/0.58%), Asymptomatic ARIA (2/1.16%), IRRs (6/3.49%), Other Reasons (2/1.16%), Other Diseases (2/1.16%) |
| 2nd | 10 (5.81%) | Symptomatic ARIA (1/0.58%), IRRs (2/1.16%), Anxiety About Side Effects (1/0.58%), Financial Burden (3/1.74%), Other Reasons (2/1.16%), Other Diseases (1/0.58%) |
| 3rd | 10 (5.81%) | IRRs (1/0.58%), Financial Burden (1/0.58%), Other Reasons (5/2.91%), Cerebrovascular Disease (1/0.58%), Dissatisfaction with Efficacy (1/0.58%), Other Diseases (1/0.58%) |
| 4th | 14 (8.14%) | Symptomatic ARIA (1/0.58%), Asymptomatic ARIA (5/2.91%), Financial Burden (1/0.58%), Other Reasons (3/1.74%), Cerebrovascular Disease (2/1.16%), Dissatisfaction with Efficacy (1/0.58%), Accident (1/0.58%) |
| 5th | 19 (11.05%) | Symptomatic ARIA (1/0.58%), Asymptomatic ARIA (2/1.16%), IRRs (2/1.16%), Financial Burden (8/4.65%), Other Reasons (3/1.74%), Dissatisfaction with Efficacy (1/0.58%), Accident (1/0.58%), Other Diseases (1/0.58%) |
| 6th | 17 (9.88%) | Asymptomatic ARIA (3/1.74%), Anxiety About Side Effects (1/0.58%), Financial Burden (7/4.07%), Other Reasons (4/2.33%), Dissatisfaction with Efficacy (1/0.58%), Accident (1/0.58%) |
| 7th | 9 (5.23%) | Symptomatic ARIA (1/0.58%), Asymptomatic ARIA (3/1.74%), IRRs (1/0.58%), Financial Burden (1/0.58%), Other Reasons (1/0.58%), Other Diseases (1/0.58%), Aβ-PET Negativity Conversion (1/0.58%) |
| 8th | 3 (1.74%) | IRRs (1/0.58%), Other Reasons (2/1.16%) |
| 9th | 5 (2.91%) | IRRs (2/1.16%), MRI Scan Intolerance (1/0.58%), Financial Burden (1/0.58%), Other Diseases (1/0.58%) |
| 10th | 3 (1.74%) | IRRs (1/0.58%), Financial Burden (1/0.58%), Other Diseases (1/0.58%) |
| 11th | 5 (2.91%) | Symptomatic ARIA (1/0.58%), Asymptomatic ARIA (1/0.58%), IRRs (1/0.58%), Financial Burden (1/0.58%), Other Reasons (1/0.58%) |
| 12th | 8 (4.65%) | Asymptomatic ARIA (1/0.58%), Anxiety About Side Effects (1/0.58%), Financial Burden (3/1.74%), Other Reasons (1/0.58%), Accident (1/0.58%), Other Diseases (1/0.58%) |
| 13th | 9 (5.23%) | Symptomatic ARIA (1/0.58%), Asymptomatic ARIA (1/0.58%), IRRs (1/0.58%), Anxiety About Side Effects (2/1.16%), Other Reasons (1/0.58%), Dissatisfaction with Efficacy (1/0.58%), Other Diseases (1/0.58%), Aβ-PET Negativity Conversion (1/0.58%) |
| 14th | 1 (0.58%) | Asymptomatic ARIA (1/0.58%) |
| 15th | 4 (2.33%) | Asymptomatic ARIA (1/0.58%), Anxiety About Side Effects (1/0.58%), Financial Burden (2/1.16%) |
| 16th | 4 (2.33%) | Asymptomatic ARIA (1/0.58%), Other Diseases (2/1.16%), Aβ-PET Negativity Conversion (1/0.58%) |
| 17th | 6 (3.49%) | Asymptomatic ARIA (2/1.16%), Financial Burden (2/1.16%), Other Reasons (1/0.58%), Dissatisfaction with Efficacy (1/0.58%) |
| 18th | 6 (3.49%) | Asymptomatic ARIA (1/0.58%), Financial Burden (2/1.16%), Other Reasons (1/0.58%), Dissatisfaction with Efficacy (1/0.58%), Aβ-PET Negativity Conversion (1/0.58%) |
| 19th | 3 (1.74%) | Symptomatic ARIA (1/0.58%), Asymptomatic ARIA (1/0.58%), Other Reasons (1/0.58%) |
| 20th | 2 (1.16%) | Financial Burden (1/0.58%), Other Reasons (1/0.58%) |
| 21st | 1 (0.58%) | Financial Burden (1/0.58%) |
| 22nd | 1 (0.58%) | Financial Burden (1/0.58%) |
| 23rd | 4 (2.33%) | Financial Burden (2/1.16%), Other Reasons (1/0.58%), Other Diseases (1/0.58%) |
| 25th | 2 (1.16%) | Symptomatic ARIA (1/0.58%), Financial Burden (1/0.58%) |
| 26th | 3 (1.74%) | Asymptomatic ARIA (1/0.58%), Financial Burden (1/0.58%), Other Reasons (1/0.58%) |
| 28th | 1 (0.58%) | Other Reasons (1/0.58%) |
| 29th | 1 (0.58%) | Other Reasons (1/0.58%) |
| 30th | 2 (1.16%) | Other Reasons (2/1.16%) |
| 32nd | 2 (1.16%) | Asymptomatic ARIA (1/0.58%), Other Reasons (1/0.58%) |
| 34th | 1 (0.58%) | Other Diseases (1/0.58%) |
| 37th | 1 (0.58%) | Other Reasons (1/0.58%) |
| 40th | 2 (1.16%) | IRRs (1/0.58%), Cerebrovascular Disease (1/0.58%) |

Abbreviations: ARIA, amyloid-related imaging abnormality; IRRs, infusion-related reactions; Aβ, amyloid-beta; PET, positron emission tomography; MRI, magnetic resonance imaging.

**eTable 8. Follow-Up Demographic and Cognitive Characteristics**

| **Variable** | **Baseline** | **3-month follow-up** | ***P* value** | **6-month follow-up** | ***P* value** | **12-month follow-up** | ***P* value** |
| --- | --- | --- | --- | --- | --- | --- | --- |
| Participants, No. | 1042 | 453 | - | 359 | - | 97 | - |
| Age, mean (SD), y | 68.93 (8.60) | 69.47 (8.34) | 0.176 | 68.38 (8.76) | 0.494 | 66.95 (9.30) | 0.080 |
| Female, No. (%) | 661 (63.44) | 278 (61.37) | 0.447 | 241 (67.13) | 0.207 | 68 (70.10) | 0.191 |
| Education, mean (SD), y | 11.35 (4.02) | 11.16 (4.19) | 0.707 | 11.33 (4.21) | 0.956 | 11.11 (4.01) | 0.547 |
| ApoE ε4 Status No. (%) |  |  | 0.004 |  | ＜0.001 |  | 0.048 |
| ε4 Noncarrier | 467 (44.82) | 219 (48.34) |  | 145 (40.39) |  | 40 (41.24) |  |
| ε4 Heterozygous | 423 (40.60) | 190 (41.94) |  | 179 (49.86) |  | 49 (50.52) |  |
| ε4 Homozygous | 93 (8.93) | 37 (8.17) |  | 32 (8.91) |  | 8 (8.25) |  |
| ε4 Unknow | 59 (5.66) | 7 (1.55) |  | 3 (0.84) |  | 0 (0.00) |  |
| MMSE score, mean (SD) | 19.94 (4.97) | 20.14 (4.89) | 0.267 | 20.81 (4.62) | 0.004 | 21.68 (4.28) | ＜0.001 |
| CDR-GS, Median (Q1,Q3) | 0.5 (0.5, 1.0) | 0.5 (0.5, 1.0) | 0.623 | 0.5 (0.5, 1.0) | 0.990 | 0.5 (0.5, 1.0) | 0.505 |
| CDR-SB score, mean (SD) | 3.82(2.81) | 3.90 (2.57) | 0.212 | 3.83 (1.95) | 0.021 | 3.56 (1.81) | 0.724 |
| ADL score, mean (SD) | 25.32(6.73) | 24.62 (5.93) | 0.159 | 25.01 (5.42) | 0.502 | 24.24 (4.47) | 0.579 |

Abbreviations: ADL, Activities of Daily Living; ApoE, apolipoprotein E; CDR-GS, Clinical Dementia Rating-Global Scale; CDR-SB, Clinical Dementia Rating-Sum of Boxes; MMSE, Mini-Mental State Examination.

P-value is relative to the baseline.

**eTable 9. Changes in Cognitive Assessment Scores From Baseline to 12-Month Follow-Up**

| **Variable** | **Follow-up** | **Total** | ***P* value** |
| --- | --- | --- | --- |
| CDR-SB change | 3-month | -0.09 (-0.19, 0.01) | 0.082 |
|  | 6-month | -0.02 (-0.18, 0.13) | 0.773 |
|  | 12-month | 0.3 (-0.05, 0.65) | 0.097 |
| MMSE change | 3-month | 0.37 (0.13, 0.62) | 0.003 |
|  | 6-month | 0.24 (-0.09, 0.57) | 0.151 |
|  | 12-month | -0.38 (-1.17, 0.41) | 0.347 |
| CDR-GS change | 3-month | 0.02 (-0.01, 0.04) | 0.162 |
|  | 6-month | 0.01 (-0.02, 0.04) | 0.412 |
|  | 12-month | 0.06 (-0.02, 0.14) | 0.145 |
| ADL change | 3-month | 0.31 (0.00, 0.62) | 0.050 |
|  | 6-month | 0.44 (0.00, 0.88) | 0.050 |
|  | 12-month | -0.22 (-0.99, 0.54) | 0.566 |

Abbreviations: ADL, Activities of Daily Living; CDR-GS, Clinical Dementia Rating-Global Scale; CDR-SB, Clinical Dementia Rating-Sum of Boxes; MMSE, Mini-Mental State Examination.

****eTable 10. Follow-Up Subgroup analyses of CDR-SB and MMSE changes by diagnostic group, sex, disease severity and Apolipoprotein E (ApoE)** ε**4 genotype****

|  |  | **CDR-SB change** | | | **MMSE change** | | |
| --- | --- | --- | --- | --- | --- | --- | --- |
|  |  | **3-month** | **6-month** | **12-month** | **3-month** | **6-month** | **12-month** |
| Diagnostic  group | BBMs-only | 0.19 (-0.23, 0.61) | -0.05 (-0.79, 0.69) | 0.9 (-1.43, 3.23) | -0.07 (-1.11, 0.96) | 0.23 (-1.34, 1.79) | -2.4 (-6.95, 2.16) |
|  | Others | -0.11 (-0.22, 0.00) | -0.02 (-0.18, 0.14) | 0.28 (-0.08, 0.64) | 0.4 (0.15, 0.65) | 0.24 (-0.1, 0.58) | -0.31 (-1.11, 0.5) |
|  | P-value | 0.172 | 0.936 | 0.602 | 0.38 | 0.985 | 0.373 |
| Sex | Male | 0.02 (-0.14, 0.19) | 0.1 (-0.17, 0.38) | 0.73 (0.09, 1.37) | 0.2 (-0.19, 0.6) | 0.06 (-0.52, 0.64) | -1.42 (-2.84, 0.01) |
|  | Female | -0.17 (-0.3, -0.03) | -0.08 (-0.28, 0.11) | 0.11 (-0.31, 0.53) | 0.48 (0.17, 0.8) | 0.33 (-0.07, 0.74) | 0.09 (-0.86, 1.04) |
|  | P-value | 0.08 | 0.282 | 0.113 | 0.279 | 0.455 | 0.087 |
| ApoE ε4 Status | ε4 Noncarrier | -0.17 (-0.32, -0.02) | -0.11 (-0.36, 0.13) | -0.05 (-0.57, 0.47) | 0.13 (-0.22, 0.48) | 0.25 (-0.27, 0.77) | -0.56 (-1.78, 0.67) |
|  | ε4 Heterozygous | -0.03 (-0.19, 0.13) | 0 (-0.23, 0.22) | 0.44 (-0.05, 0.93) | 0.72 (0.34, 1.1) | 0.21 (-0.26, 0.68) | -0.33 (-1.47, 0.81) |
|  | ε4 Homozygous | 0.06 (-0.3, 0.42) | 0.27 (-0.26, 0.8) | 1.71 (0.23, 3.19) | 0.15 (-0.71, 1.01) | 0.26 (-0.84, 1.36) | 0.47 (-2.71, 3.65) |
|  | P-value | 0.33 | 0.418 | 0.06 | 0.07 | 0.991 | 0.829 |
| Disease severity | Mild | -0.1 (-0.21, 0.01) | 0 (-0.16, 0.16) | 0.31 (-0.05, 0.66) | 0.43 (0.18, 0.69) | 0.15 (-0.18, 0.48) | -0.41 (-1.22, 0.39) |
|  | Moderate to severe | 0.1 (-0.45, 0.66) | -0.75 (-1.72, 0.22) | -0.7 (-4.51, 3.1) | -0.51 (-1.67, 0.66) | 1.74 (-0.13, 3.61) | 1.29 (-4.27, 6.85) |
|  | P-value | 0.486 | 0.138 | 0.602 | 0.127 | 0.101 | 0.548 |

Abbreviations: ApoE, apolipoprotein E; BBMs, blood-based biomarkers; CDR-SB, Clinical Dementia Rating-Sum of Boxes; MMSE, Mini-Mental State Examination.

**eFigure 1. The flow chart of the study**

**eFigure 2. Reasons for Treatment Discontinuation**

Abbreviations: ARIA, amyloid-related imaging abnormality; Aβ, amyloid-beta; PET, positron emission tomography; MRI, magnetic resonance imaging.
